# Determining the Photoisomerization Quantum Yield of Photoswitchable Molecules in Solution and in the Solid State

**DOI:** 10.1038/srep41145

**Published:** 2017-01-24

**Authors:** K. Stranius, K. Börjesson

**Affiliations:** 1 Department of Chemistry and Molecular Biology, University of Gothenburg, Kemigården 4, 412 96 Gothenburg, Sweden

## Abstract

Photoswitchable molecules are able to isomerize between two metastable forms through light stimuli. Originally being studied by photochemists, this type of molecule has now found a wide range of applications within physics, chemistry and biology. The extensive usage of photochromic molecules is due to the two isomers having fundamentally different physical and chemical properties. The most important attribute of a photoswitch is the photoisomerization quantum yield, which defines the efficiency of the photoisomerization event. Here we show how to determine the photoisomerization quantum yield in the solid state and in solution when taking thermal processes into account. The described method together with provided software allows for rapid and accurate determination of the isomerization process for this important class of molecules.

Photochromism is defined as the light induced reversible transformation between two chemical species having different absorption spectra^1^,^2^. The first example of a photochromic molecule was made in the late 19^th^ century, and today a vast number of photoswitchable molecules exist^3^. These have been used over all natural science disciplines, where the different properties of the two photoisomers are exploited. For instance, the conformational change between two photoisomers is used in the retina to provide vision, and has been used for photo-reversible DNA and protein binding^4^,^5^. Also photoactivated drugs have been demonstrated^6^,^7^. The change in optical properties has been used in optical memories and logic gates^8^,^9^. The change in polarity has been exploited to photoswitch surface hydrophobicity^10^,^11^. Furthermore, the energy difference has been used to collect and store solar energy^12^,^13^, and the difference in the LUMO or HOMO levels has been used for photoswitchable electronics^14^,^15^.

There is no photoswitchable molecule that excels in all types of applications, but the following families of molecules are most commonly used, all having different kinds of mode of action. The diarylethene (dithienylethene) was developed in the 1980’s by Masahiro Irie^16^. In this very useful class of photoswitchable molecules a photoinduced pericyclic reaction closes or opens the central ring (Fig. 1a), changing both the electronic properties as well as to the flexibility between the two isomers. In spiropyrans a photoinduced heterolytic bond cleavage leads to the formation of the flat and zwitterionic merocyanine isomer (Fig. 1b)^17^. In azobenzenes the central double bond is in the excited state weakened, leading to free rotation and thus conversion between the cis/trans isomer (Fig. 1c)^18^. In fulgides the central ring unit opens or closes through a pericyclic reaction (Fig. 1d)^19^. Lastly in this short summary of common photoswitchable molecules, norbornadienes isomerize to the highly ring strained high energy quadricyclane by a photoinduced [2 + 2] cycloaddition (Fig. 1e)^20^.

The single most important system property of a photochromic molecule is the photo induced reaction that interconverts the two isomers (the photoisomerization quantum yield). Depending on mode of action and spatial requirements, this value will be affected by environmental factors such as viscosity, temperature, and polarity of the solvent. We here describe how the efficiency of this reaction can be determined both in solution and in the solid state, and how thermal relaxation can be taken into account in the analysis. A kinetic model of the isomerization process is described and used to determine the photoisomerization quantum yield in a set of practical examples, both in solution and in the solid state. Furthermore, an easy to use program that can fit experimental data to the kinetic model is provided (Supplementary Software S1 and S2). The program numerically solves an ordinary differential equation in each iteration step in the fitting procedure, and can thus unlike earlier work^21^,^22^, fit data to theory without the need of analytical solutions or mathematical simplifications. The intention here is to give a comprehensive theoretical background combined with all needed practical information for determining photochemical quantum yields in solution as well as solid state and provide the necessary software needed for data analysis.

## Results and Discussion

### The rate equations describing the isomerization process

The rate equations describing the photoisomerization process of a photochromic molecule have earlier been thoroughly discussed^1^,^2^,^3^,^23^. Here we first give a summary of the rate equation describing the system, followed by showing how these equations can be used to determine the photoisomerization quantum yield in solution for simple cases. Finally we will explore two more demanding cases: (1) When there is a need to compensate for thermal processes, and (2) when the photochromic molecule is in the solid state.

**Figure 2:**
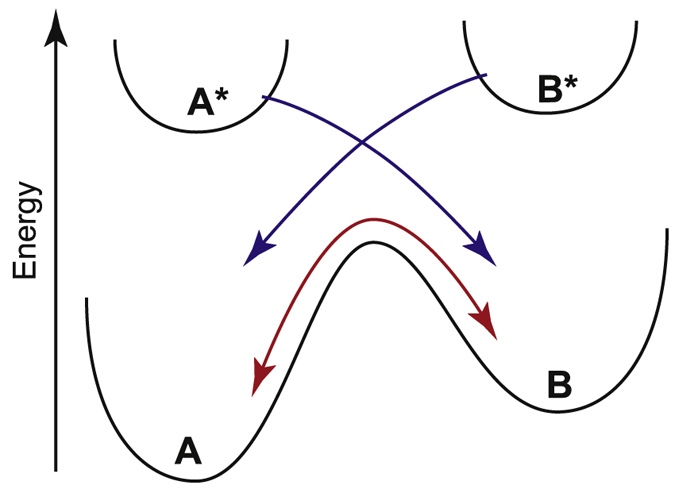
The two photoisomers, A and B, can convert to one another by light (blue arrows) or by a spontaneous thermal induced process (red arrow).

A photochromic molecule can in most cases be viewed as a two state system, A and B, where at thermal equilibrium A is the most stable isomer. However, in presence of light A will isomerize to B (Fig. 2, Equation 1).


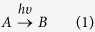


The change in concentration of A is proportional to the quantum yield of photoisomerization (*ϕ*_*A*_, unitless), the photon flux (*I*, s^−1^) and the fraction of photons absorbed by A (β, unitless).


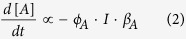


As the concentration of B is building up, B will photoisomerize back to A, provided that B absorbs light at the irradiation wavelength and the photoisomerization quantum yield for the reverse process is non zero.


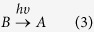



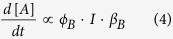


The isomerization from B to A can also be spontaneous. The thermally induced spontaneous isomerization is a first order process (Supplementary Note). Thus, overall three rates are needed to completely explain the isomerization from A to B (Fig. 2):


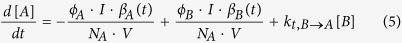


where *N*_*A*_ is Avogadro’s number, *k*_*t*_ is the rate constant for spontaneous back conversion, and *V* the volume of the sample (in dm^3^). In the following sections Equation 5 will be used to determine the photochemical quantum yield in a number of special cases, and methods for determining the concentration of A and B, the photon flux, and fraction of photons absorbed by A and B will be discussed.

### Photoisomerization in solution

A great majority of reported photoisomerization quantum yield measurements have been performed in solution. To be able to employ equation 5 on a sample in solution, the concentrations of A and B needs to be homogenous within the sample at all times. This requirement can only be kept with fast enough stirring. What fast enough is depends on many factors, such as the photon flux and size of light beam. Also in ideal cases, no side products occur in the photoisomerization reaction and A is cleanly converted into B. Thus, as long as the initial concentration is known (*C*_*0*_), it is sufficient to either determine the concentration of A or B, or the mole fraction of A and B (*χ*) to follow the reaction.


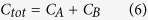



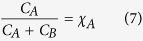


Many techniques, such as NMR, chromatography and UV/Vis spectroscopy can be used to determine the concentration of A and B. However, UV/Vis spectroscopy is the method of choice since it not only gives information on the concentration of a species, but also gives information on the amount of light that is absorbed. In a spectrophotometer, the measured value is the total absorbance of the sample and it is therefore important to express all concentrations as a function of the total absorbance. Absorbance is an additive property, such that:


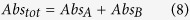


By using the Beer-Lambert law and Equations 6 and 8, the concentration of A can be expressed through the total absorbance and concentration:


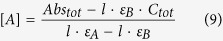


where *ε* is the molar absorptivity (in M^−1^ cm^−1^) and *l* is the path length (in cm^−1^). The next variable that needs to be determined in Equation 5 is the fraction of light absorbed, β. The fraction of absorbed light can be divided into two regimes: (1) All incident light is absorbed by A; and (2) some light passes through the sample, and/or species B absorb some light.

### Regime 1: Total absorption regime

Regime 1 is the easiest to analyze, and it corresponds to a situation where the absorbance of species A is above 2 during the whole experiment time (at least 99% of the photons are then absorbed) and at the same time species B does not absorb at all during the experiment. We define species B not to absorb when:


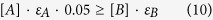


The drawback of regime 1 is that it limits the concentration ranges to be used, since relatively concentrated samples are needed. However, it is the method of choice if NMR or chromatography is wanted as analysis methods (both having the limitation that only one data point can be obtained per sample). UV/Vis spectroscopy can be used if a spectral region having low enough absorption is found at the relatively high concentrations used. The advantage of working in regime 1 is, provided the thermal back reaction is slow, that Equation 5 can be simplified and analytically integrated to give a linear dependence between the concentration of A and the irradiation time (*t*_*irr*_). The photochemical quantum yield can be calculated by a linear fit to acquired data (Supplementary Fig. S1), which slope then is identified by Equation 11:


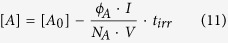


### Regime 2: Low absorption regime

In regime 2, the fraction of photons that actually are absorbed by A and B needs to be determined, which results in a differential equation with no analytical solution (vide infra). However, today the required computer power to solve differential equations numerically is small, and it is well worth doing, since it allows for a general method that can be used for most types of molecules. The amount of photons absorbed (β) by A (and/or B) is determined by recording the absorbance at the wavelength of irradiation. Importantly, it is here assumed that the light of irradiation is collimated. Non-collimated light will give a longer path length through the cuvette, which results in more light being absorbed than taken into consideration in Equation 12.


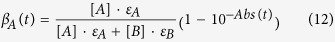


In regime 2 it is possible to determine the photoisomerization quantum yields at lower concentrations than in regime 1, and it is also possible to take into account all photoinduced and thermal processes of the system. However, it requires that the molar absorptivity of A is different from B at the wavelength of irradiation, and that the molar absorptivity of A is known (the molar absorptivity of B needs to be known or to be negligible). For a pure compound, determining the molar absorptivity is trivial *i.e.* requiring only recording the absorption spectrum of a sample of known concentration. If only the low energy species (A) is possible to obtain in pure form, it is possible to mathematically construct the molar absorptivity spectrum of B by first recording the absorption spectrum of pure A, followed by irradiation until steady state occurs and then recording the absorption spectrum again. A complementary technique (such as NMR) is then used to determine the molar ratio of A and B at steady state, which enables the absorption spectrum of B to be calculated.

In many cases, it is safe to assume that the thermally activated back isomerization is slow and that only A absorbs light at the wavelength of irradiation. Thus, the only process that needs to be taken into account is the photoinduced one from A to B (Fig. 2) and Equation 5 can be simplified to:


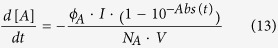


**Figure 3:**
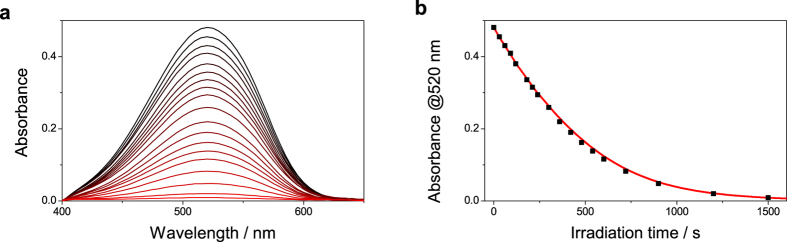
**(a)** Absorbance of the closed form of the fulgide Aberchrome 670 dissolved in toluene after increasing amount of irradiation. **(b)** Absorbance at 520 nm (black squares) as a function of irradiation time. The red solid line shows a fit of the experimental data to Equation 13.

In which the concentration of A easily can be determined using the Beer-Lambert law. Important to note here is that the concentration of A and the ratio of light absorbed by A both can be determined by the absorbance value at the wavelength of irradiation. UV/Vis spectroscopy is thus a very convenient technique to perform photoisomerization quantum yield determinations. From Equation 13 and onwards it is assumed that recorded absorbance values are taken at the wavelength of irradiation and that the path length of the light of irradiation is the same as the path length in the absorbance measurement (in Supplementary Software S1 and S2 this is assumed to be 1 cm). Furthermore, if thermal processes are slow, only the actual irradiation time needs to be taken into account. Thus, the time between irradiation and analysis is of no consequence, which makes it possible to use separate experimental setups for irradiation and probing. Figure 3 show the absorbance of the closed form of the fulgide Aberchrome 670 as a function of irradiation time together with a fit using Equation 13. The photoisomerization quantum yield of Aberchrome 670 is known, and in this case the photon flux was used as a fitting parameter in Equation 13. That is, Equation 13 can be used either to determine the photochemical quantum yield or to determine the photon flux using a chemical actinometer (See Supplementary Note, Supplementary Fig. S1, and Supplementary Table S1 for a discussion on chemical actinometry).

If both A and B absorbs light at the wavelength of irradiation but thermal reactions are slow, Equation 5 can be simplified to:


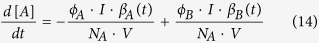


Assuming that only A is present at start, the total absorbance relates to the concentration of A through Equation 9, and the fraction of absorbed light through Equation 12. However, fitting the quantum yields of both A and B simultaneously is often not done. Instead, the photoisomerization quantum yield of either A or B is usually first obtained by either working in absorption regime 1 (Equation 11) or at a wavelength were only one species absorbs (which exists for many photoswitches; Equation 13). When the quantum yield of A (or B) is known, Equation 14 can be used to fit the quantum yield of B (or A). However, if A and B have high enough photo- and thermal stability, a solution can be irradiated until a steady state is reached.


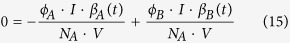



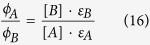


At steady state, the molar ratio of A and B is easily determined using for instance NMR, which enables *ϕ*_*B*_ to be calculated if *ϕ*_*A*_ is known (Equation 16). In practice, an isosbestic point is often chosen as the wavelength of irradiation. By doing so, the determined photoisomerization quantum yield is not affected by any uncertainty in the values of the molar absorptivities, since those are by definition exactly the same at the isosbestic point.

### Taking thermal processes into account

**Figure 4:**
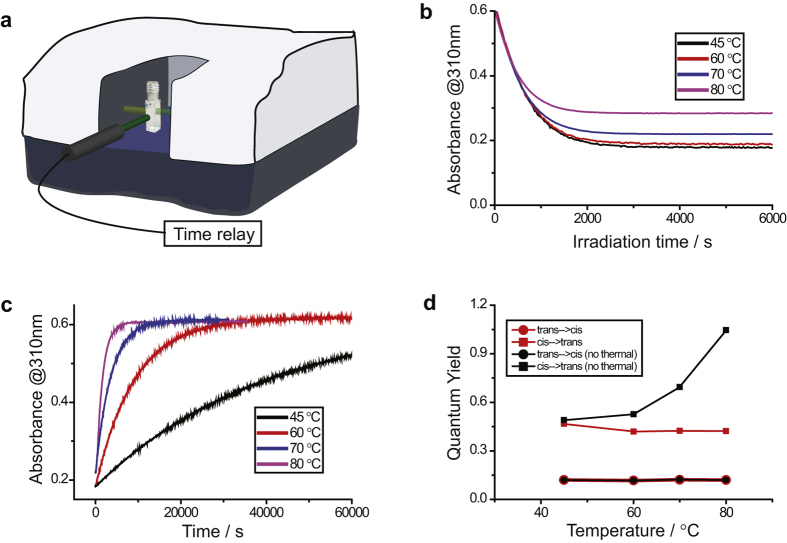
**(a)** Schematic illustration of a sample irradiated inside a spectrophotometer, with a time relay controlling the lamp. **(b)** Absorption of azobenzene vs time starting from pure trans form while simultaneously irradiation the sample. **(c)** Absorption of azobenzene vs time starting from a cis/trans mixture **(d)**. Photoisomerization quantum yields of azobenzene in decane solution as a function of temperature (red: calculated using Equation 5, black: calculated using Equation 14, neglecting the thermal process).

If the spontaneous back-conversion from B to A occurs on a timescale similar to the time it takes to conduct the experiment, it needs to be taken into account. What is important to keep in mind for this case is that the thermal back reaction depends on elapsed time, but the photoisomerization reaction depends only on the total time of irradiation. The easiest way to merge these two timescales is by using a cuvette with four polished sides that is irradiated normal to the probe beam inside a spectrophotometer (Fig. 4a). The measurement is conducted so that the light source is repeatedly switching on/off at fixed time intervals and the absorbance of the sample is measured when the light source is switched off as to avoid scattered light during measurement. The irradiation time is converted to real time by averaging the photon flux over an on/off period. Many light sources can be programmed to switch on and off or be externally triggered. However, less sophisticated light sources can be switched on/off by the use of an electric shutter, controlled by an inexpensive time delay relay or by a LabVIEW script. Figure 4a show a schematic picture of a setup inside a spectrophotometer. A time delay relay is used to switch a light source on/off, and synchronization between the relay and spectrophotometer is done manually. Using this approach, it is not the manual synchronization between the spectrophotometer and lamp that puts a limit on how fast rates of back conversion that can be taken into account, instead it is the assumption that the concentration of A (and B) is homogeneous in the cuvette. This assumption puts a practical limit of the irradiation time between two data points to a couple of seconds, again depending on stirring speed and size of light beam. An alternative setup would be to use a strong collimated light source that is used both to irradiate and to probe the transmittance of the sample, in analogy to earlier reports on photoisomerization quantum yield measurements in films^24^,^25^,^26^.

Azobenzene is one of the oldest photoswitches known, but new derivatives thereof are still being developed^27^,^28^,^29^,^30^. The low energy state of azobenzene is the trans-isomer and the high energy is the cis-isomer, which spontaneously isomerizes back to the trans isomer at ambient temperatures. At higher temperatures the rate of the thermal back conversion is increased and is on the same timescale as the photoisomerization process. Thus, the thermal back conversion needs to be taken into account when analyzing the photoisomerization quantum yield. Also, both the cis and trans form of azobenzene absorb light at the same wavelength range, giving rise to a photostationary state at long irradiation times. Figure 4b–d show photoisomerization, thermal back conversion and photoisomerization quantum yields of azobenzene dissolved in decane at various temperatures. The effect of the thermal back reaction on the photostationary state can be seen in Fig. 4b. The trans isomer has a higher molar extinction coefficient than the cis isomer, and thus the increased rate of the thermal back reaction at higher temperatures results in a steady state having a higher degree of the trans isomer. The obtained steady state is thus better described as a photo*thermal*stationary state rather than as a photostationary state. Figure 4d shows how the fitted quantum yield for the forward trans-cis isomerization reaction is less sensitive on the thermal back reaction than the back cis-trans isomerization reaction. The reason for this is because the fitted quantum yield for the forward reaction is governed by the initial slope in Fig. 4b. The relative difference in rates between the thermal reaction and the trans to cis photoisomerization reaction is large at short timescales (Supplementary Fig. S2) and thus the negligence of the thermal process has little consequence on the fitted quantum yield. However, the fitted quantum yield for the cis to trans photoisomerization is highly dependent on the thermal back reaction. This because the fitted value for the photoisomerization quantum yield for the back reaction, to a high degree, is governed by the mole fractions of the cis and trans isomers at the photothermalstationary state. As seen in Fig. 4d, when taking thermal isomerization into account, the photoisomerization quantum yield of the cis to trans process is constant over the measured temperature range. Whereas the negligence of the thermal process leads to overestimated quantum yields. This result can again be rationalized with relative rates. The relative rates for the cis to trans photo- and thermal reactions is on the same order of magnitude at the photothermalstationary state (Supplementary Fig. S2). Thus, neglecting the thermal process in the analysis greatly overestimates the photoisomerization quantum yield for the cis to trans reaction. Therefore, when determining photoisomerization quantum yields, it is always important to be aware of thermal relaxation processes. However, as shown here, the thermal relaxation process can be accounted for, as long as the time of irradiation and elapsed time is correlated in the performed experiment.

### Photoisomerization in solid films

Literature on photoisomerization quantum yields, measured in the solid state exist but are scarce^31^,^32^,^33^,^34^,^35^,^36^, most likely due to earlier published procedures requiring significant knowledge of spectroscopy and/or optics^24^,^25^,^26^,^37^. However, it is not at all impossible, and only a few modifications and assumptions are needed to adapt Equation 13 to the solid state. In the following section it is assumed that the molar absorptivity in the solid state is known, and that the Beer-Lambert law is applicable. In some cases it can be difficult to determine the molar absorptivity with high accuracy in a solid film, but it is always possible to compare the envelope of the absorption spectrum in the solid state vs solution, and thus show that no major changes occurs when going from solution to the solid state.

**Figure 5:**
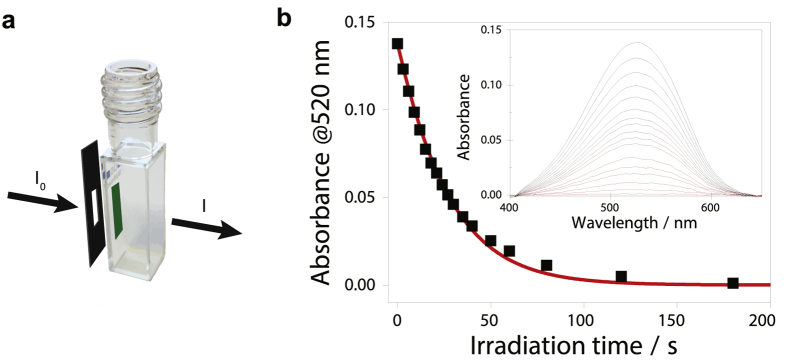
**(a)** Schematic picture of a cuvette having a thin film supported by a glass substrate on the inside and a mask with defined area on the outside. **(b)** Absorbance at 520 nm (black squares) of the closed form of the fulgide Aberchrome 670 in polystyrene as a function of irradiation time. The red solid line shows a fit of the experimental data to Equation 18. The full absorbance spectra are shown in the insert.

If it is assumed that a thin film is irradiated homogenously (*i.e.* the light is collimated and the photon flux is uniformly distributed over the whole film area), and that the film thickness is *d* and the path length of light irradiation is *l*, then Equation 13 can be rearranged to:


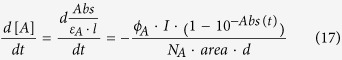


where the volume of the sample has been replaced with the film area multiplied with the film thickness. When both irradiation and probing of the film is done normal to the film, the film thickness equals to the path length in the Beer-Lambert law, and Equation 17 can be reduced to give:


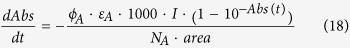


where the factor 1000 is due to a conversion of units in order to have *l, d*, and *area* all being expressed in cm (or cm^2^) to allow for the mathematical simplification. We have found that the easiest method of doing this type of experiment is by pasting a mask onto a cuvette. The mask is typically made of a non-transparent self-adhesive material in which a hole has been cut (typically 4*4 mm; the exact size of the hole can for instance be determined by weighing the cut piece and using the area density of the material to calculate the area). By determining the photon flux through the mask (see Supplementary Note, Supplementary Fig. S1, and Supplementary Table S1 for details on photon flux determination through chemical actinometry), an accurate value of the area normalized photon flux can be determined. A thin film of A, either pristine or in a polymer matrix, supported by for instance a glass substrate is then placed on the inside of the cuvette in such manner that the film covers the whole area of the hole in the mask (Fig. 5a). By determining the absorbance of the film through the mask as a function of irradiation time, the quantum yield of A can be determined through Equation 18.

Figure 5b shows the absorbance as a function of irradiation time of a thin film of Aberchrome 670 in a polystyrene matrix. Equation 18 was used to fit the absorbance data giving a photoisomerization quantum yield of 27%. To compare, the photoisomerization quantum yield of Aberchrome 670 in toluene solution is 30%^38^. The small difference going from solution to the solid state can be rationalized by the reaction mechanism. Fulgides isomerizes through a pericyclic reaction, which requires a relatively small spatial rearrangement between the starting material and product. Thus, the photoisomerization process is not affected by the rigidity (viscosity) of the matrix used.

The here described methods gives reproducible photoisomerization quantum yields in the solid state. However, of importance is the film homogeneity. Films should have low tendency to scatter light. Also, variations in film thickness need to be avoided since it would result in erroneous calculation of the isomerization quantum yields. Therefore, the preferred film deposition technique is spin-coating rather than drop-casting. Spin-coating generally results in films having a consistent thickness over a large area. It should be noted that an interesting phenomena occurs in thick films. Since diffusion inside the film is negligible, in high absorbance films, the light intensity will decrease with increased penetration depth, causing a concentration gradient of A and B in the film. It is possible to take this concentration gradient within the film into account. It is done by adding a numerical integration step along the direction of the film thickness in the analysis. For more information about this step, the reader is referred to earlier work^24^,^25^,^26^,^37^. The concentration gradient is not taken into consideration in the discussed method and is of no consequence as long as only A absorbs light. Furthermore, the effect can be completely removed if films with low initial absorbance (Abs < 0.05) are used.

Photochromic crystals based on diarylethene derivatives has received considerable attention in recent years^16^. The photoisomerization quantum yield of photochromic crystals can in some cases be determined using the described method, prerequisite that the crystal is sufficiently large and have a uniform thickness. However, three attributes complicates the analysis of the crystal case compared to the case when the photochromic molecule is dissolved in a solid matrix: (1) The absorbance of a crystal is anisotropic. Thus, it is important to either use unpolarized light for irradiation and probing, or use polarized light for irradiation and probing with the same angle between the plane of polarization and crystal axis. (2) The molar extinction coefficient is normally determined at low concentrations assuming isotropic orientation. Determination of the molar extinction coefficient for each crystal using the relevant orientation of the probe light is thus necessary, both for taking into account molecular orientation within the crystal and also spectral shifts of the absorbance spectrum due to exciton coupling^39^. (3) In a crystal, as the photoisomerization prolongs, the local environment for each molecule changes. If isomerization starts with all molecules in the form of isomer A, as the isomerization continues, the concentration of isomer B is gradually increasing. The photoisomerization quantum yield (and also the absorbance spectra of A and B in the crystal) depends on the local environment of the individual molecule. Thus, it differs between the beginning and end of the photoisomerization process, which severely complicates the analysis. Therefore, the described method can be used to determine the photoisomerization quantum yield in photochromic crystals only for the cases when only one of the isomers selectively can be excited and the concentration of that species can be regarded as low (ensuring similar environment throughout the experiment and a low total absorbance).

## Conclusion

A detailed procedure for determining photoisomerization quantum yields of photoswitchable molecules is presented. The method is based on numerical integration of a kinetic model that takes photo as well as thermal processes into account. Furthermore, the method is expanded to also cover reactions in the solid state. To demonstrate the applicability, the photoisomerization quantum yield of azobenzene at different temperatures is determined, showing the need of taking thermal processes into account in the analysis at elevated temperatures. Also, the photoisomerization quantum yield of Aberchrome 670 in a polystyrene film is determined.

## Methods

### Instrumentation

All chemicals were purchased from commercial suppliers and used without further purification. Samples were irradiated using a Spex Fluorolog spectrofluorometer (JY Horiba) or a M310L3 LED (THORLABS) equipped with a collimator lens. Absorbance was measured using a Cary 4000 (Varian), Cary 60 (Agilent), or a Lambda 650 (PerkinElmer) spectrophotometer. Films of Aberchrome 670 were made by spin-coating (3,000 rpm, Laurell Technologies) a chloroform solution containing polystyrene (63 mg/ml) and Aberchrome 670 (6.5 mg/ml).

### Analysis

Molar absorptivity values for trans and cis azobenzene was set to 21,000 M^−1^ cm^−1^ and 1,400 M^−1^ cm^−1^, respectively^40^. The photon flux for the azobenzene measurements was determined using the method reported herein using earlier reported photoisomerization quantum yield (*ϕ*_*trans*→*cis*_ = 0.11) for the trans to cis photoisomerization process at room temperature^40^. Analysis of experimental data was done by fitting to respective equation using provided program written in MatLab (Supplementary Software S1 and S2). Supplementary Software S1 is compiled using MatLab R2013b (64-bit) on Windows 7, and Supplementary Software S2 is compiled using MatLab R2015b (64-bit) on OS X Yosemite. Both versions work as standalone program, without the need of a MatLab license. The program uses the method of least squares to find best fit to experimental data. In each iteration step, the chosen differential equation is solved using the *ode15s* solver (an intrinsic function within MatLab), which is a numerical ode solver specialized on stiff odes. The *ode15s* solver is faster than non-stiff solvers such as the ode45 solver on the non-oscillating type functions used here^41^. It should also be stressed that the residual of the fit always should be manually examined, as to ensure that no systematic errors are present. The input data for the software is a text file containing two columns separated by tabs. The first column contains irradiation times (in s), and the second contain absorbance values taken at the wavelength of irradiation. The software assumes that a 1*1 cm cuvette was used. The value of the quantum yields and photon fluxes are displayed within the program, and best fit to experimental data is saved as dataoutfit in the same directory as from where loaded data was taken. The saved file contains four columns representing time, fits for the concentration of A and B, and a fit for the total absorbance of the sample.

## Additional Information

**How to cite this article**: Stranius, K. and Börjesson, K. Determining the Photoisomerization Quantum Yield of Photoswitchable Molecules in Solution and in the Solid State. *Sci. Rep.* **7**, 41145; doi: 10.1038/srep41145 (2017).

**Publisher's note:** Springer Nature remains neutral with regard to jurisdictional claims in published maps and institutional affiliations.

## Supplementary Material

Supplementary Information

Fitting Programme

Fitting Programme

## Figures and Tables

**Figure 1:**
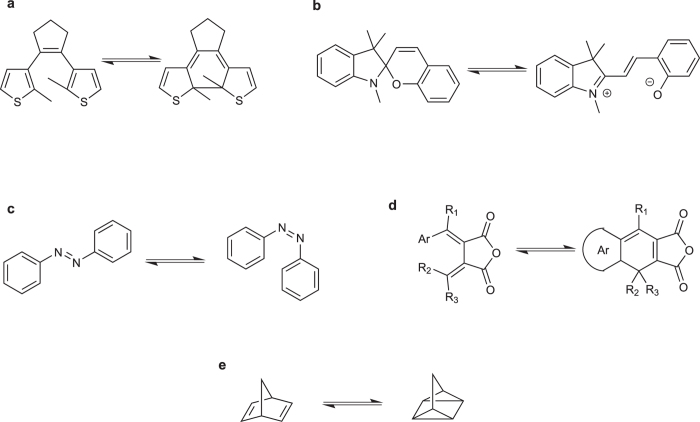
Examples of photochromic molecules. **(a)** Diarylethene (dithienylethene), (**b**) Spiropyran/merocyanine, **(c)** Azobenzene, **(d)** Fulgide (for the fulgide used in this study Ar = 2,5-dimethylfuran-3-yl, R_1_ = methyl, and R_2_ and R_3_ are both part of an adamantane unit), and **(e)** Norbornadiene/quadricyclane.
