# Supplementary material for: Determining the Photoisomerization Quantum Yield of Photoswitchable Molecules in Solution and in the Solid State
Source: Sci Rep. 2017 Jan 24;7:41145. doi: 10.1038/srep41145 (PMC5259717; doi:10.1038/srep41145)
Supplement: Supplementary Information [file srep41145-s1.pdf]

Supplementary Information

Determining the Photoisomerization Quantum Yield of Photoswitchable Molecules in Solution and in the Solid State

K. Stranius and K. Börjesson*

Department of Chemistry and Molecular Biology, University of Gothenburg, Kemigården 4, 412 96 Gothenburg, Sweden

*Correspondence to: karl.borjesson@gu.se

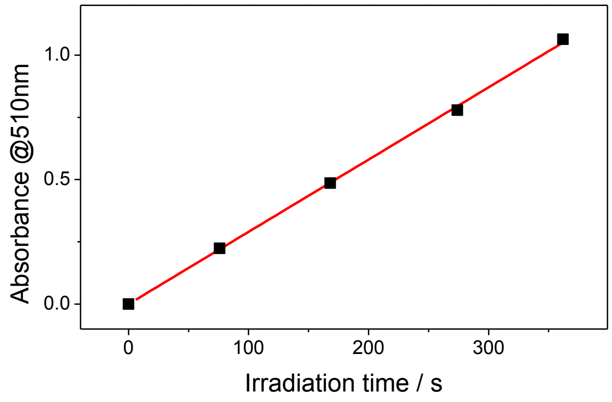

Supplementary Figure S1 *Absorbance of the tris-phenanthroline iron (II) complex as a function of irradiation time of potassium ferrioxalate (black squares). The red line shows a linear fit, which is used to calculate the photon flux through Supplementary Equation 5.*

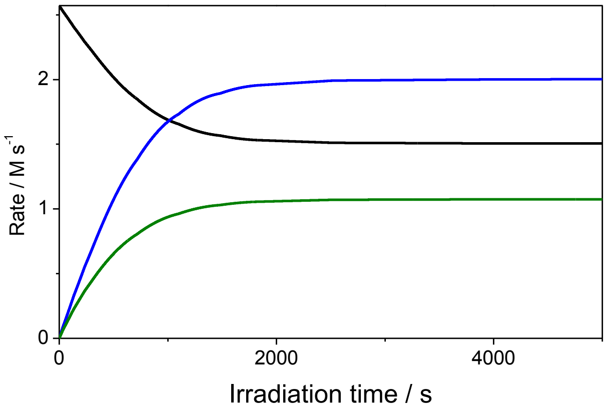

Supplementary Figure S2 *Rates of trans-cis photoisomerization (black), cis-trans photoisomerization (blue) and thermal cis-trans isomerization (green) as a function of irradiation time for* azobenzene dissolved in decane at 80°C.

Supplementary Table S1 *Photochemical quantum yield (ϕ) for the production of ferrous ions from ferrioxalate decomposition by irradiation at specific wavelengths.*

Irradiation wavelength (nm)	ϕ [a]
254	1.25
313	1.24
365.6	1.21
405	1.14
436	1.11

a) from ref 1.

Supplementary Note: The rate of thermal isomerization

The rate of thermal isomerization is a first order process and by observing the change in concentration of A (or B) in absence of light with respect to time, the rate constant (k_t) of the thermal isomerization can easily be determined (Supplementary Equation 1). The activation energy (E_A) for the thermally induced isomerization is an important system property of a photoswitch. The activation energy can be determined by observing the dependence of the temperature on k_t. The Arrhenius equation (Equation 2) is then used to calculate E_A:

$$[B] = [B_0] \cdot e^{-k_{t,B\rightarrow A} \cdot t} \qquad \text{Supplementary Equation 1}$$

$$k_t = A \cdot 10^{-E_A/RT} \qquad \text{Supplementary Equation 2}$$

Where *A* is a pre-exponential factor, *R* is the universal gas constant, and *T* is the temperature (in Kelvin).

Supplementary Note: Determination of the photon flux (I)

In the described method of determining photoisomerization quantum yields, it is assumed that the light source is monochromatic with a collimated beam profile. This can of course be achieved with a laser, but also light emitting diodes or broadband light sources with monochromator optics equipped with a collimating lens works fine. All glass surfaces reflect light to a various degree depending on material and angle of incidence, making photon flux determination with power meters non ideal. The most widely acknowledged method of determining the photon flux inside a cuvette most accurately is therefore chemical actinometry. In chemical actinometry a standard (a photochemical reaction with known quantum yield) is used as a reference to determine the photon flux. Although very simple in theory, it puts some high demand on the molecule in question. Preferably, the actinometer should be photostable and insensitive to factors such as irradiation wavelength, temperature etc., but in reality compromises need to be done since no ideal molecules exist. Many molecules have been proposed as chemical actinometers (and in principal all molecules with a known photochemical quantum yield can be used) and for a detailed list the reader is referred to earlier literature.[2,3]

In our laboratory, we are most commonly using ferrioxalate or the fulgide Aberchrome 670, depending of irradiation wavelength. The ferrioxalate method, originally described by Parker and Hatchard,[1,4] is the most commonly used chemical actinometer. Although it is a little labor intensive to use it produces excellent results and is generally recommended. The method is performed in the dark and is based on the photochemical degradation of ferrioxalate to Fe^{2+}:

$$Fe(C_2O_4)_3{}^{3-} \xrightarrow{hv} Fe^{2+} + C_2O_4{}^{\cdot-} + 2C_2O_4{}^{2-} \qquad \text{Supplementary Equation 3}$$

$$Fe(C_2O_4)_3{}^{3-} + C_2O_4{}^{\cdot-} \xrightarrow{\Delta} Fe^{2+} + 2CO_2 + 3C_2O_4{}^{2-} \qquad \text{Supplementary Equation 4}$$

The Fe^{2+} ions are complexed with phenanthroline, and the concentration of the tris-phenanthroline complex is determined using absorbance (ε_{510nm}=11,100 M^{-1} cm^{-1}). We use a slightly modified standard protocol,[5] in which first a known volume (V_1, typically 2ml) of ferrioxalate (30 mM in 0.2 N H_2SO_4) is irradiated under stirring. An aliquot of the irradiated volume (V_2, typically 0.5 ml) together with buffer (1.2 M NaAc + 0.72 N H_2SO_4, 1 ml), and phenanthroline (6 mM, 2 ml) is diluted (V_3, typically to 25 ml) and left to react for 1 h, where after the absorbance of the tris-phenanthroline complex is recorded at 510 nm. This procedure is repeated using different irradiation times to create a plot of absorbance as

a function of irradiation time (Supplementary Fig. 1). The plot should be linear, (deviations from linearity can for instance depend on depletion of ferrioxalate) and the slope is used to calculate the photon flux (I) through Supplementary Equation 5 which is a modified version of Equation 11 taking dilution factors into account:

$$I = slope \cdot \frac{V_1 \cdot V_3}{V_2 \cdot \varepsilon_{510nm} \cdot l \cdot \phi} \qquad \text{Supplementary Equation 5}$$

Where l is the path length of the cuvette and ϕ is the photochemical quantum yield of the process. A selection of recommended values of ϕ is found in Supplementary Table 1.

Ferrioxalate only absorbs light in the UV and blue part of the spectrum. In the visible part, we have had good experience in using the closed form of fulgides as actinometers (Fig. 1). The fulgide Aberchrome 670 is commercially available and upon irradiation at 366 nm it isomerizes to a thermally stable highly colored ring closed form (λ_{max}=520 nm, ε_{520nm}=5,400 M^{-1} cm^{-1} in toluene).[6,7] The back conversion of Aberchrome 670 can be used to determine the photon flux by monitoring the absorbance as a function of irradiation time and fitting the obtained data to Equation 13 (Fig. 3). The photoisomerization quantum yield of Aberchrome 670 is both temperature and wavelength dependent and Supplementary Equation 6 relates ϕ to the used experimental conditions (valid within the temperature range 22-80 °C).[6]

$$\phi = 0.4326 - (3.285 \cdot \lambda - 16.4 \cdot T) \cdot 10^{-4} \qquad \text{Supplementary Equation 6}$$

Where λ is the irradiation wavelength in nm, and T is the temperature in Celsius. Aberchrome 670 can be used in the 460-580 nm wavelength range, and provides a rapid and convenient method of determining the photon flux. Other fulgides used as actinometers include Aberchrome 540 nm.[8,9]

Supplementary References

1 Hatchard, C. G. & Parker, C. A. A new sensitive chemical actinometer .2. Potassium ferrioxalate as a standard chemical actinometer. *Proc. R. Soc. A* **235**, 518-536 (1956).

2 Kuhn, H. J., Braslavsky, S. E. & Schmidt, R. Chemical actinometry. *Pure Appl. Chem.* **76**, 2105-2146 (2004).

3 Montalti, M., Credi, A., Prodi, L. & Teresa Gandolfi, M. *Handbook of photochemistry* 3 edn, (CRC press, 2006).

4 Parker, C. A. A new sensitive chemical actinometer .1. Some trials with potassium ferrioxalate. *Proc. R. Soc. A* **220**, 104-116 (1953).

5 Fischer, E. Ferri-oxalate actinometry. *EPA Newsletter*, 33-34 (1984).

6 Glaze, A. P., Heller, H. G. & Whittall, J. Photochromic heterocyclic fulgides .7. (e)-adamantylidene- 1-(2,5-dimethyl-3-furyl)ethylidene succinic anhydride and derivatives - model photochromic compounds for optical-recording media. *J. Chem. Soc.-Perkin Trans. 2*, 591-594 (1992).

7 Yokoyama, Y. *et al.* Effects of steric bulkiness of substituents on quantum yields of photochromic reactions of furylfulgides. *Bull. Chem. Soc. Jpn.* **67**, 3297-3303 (1994).

8 Guo, Z. X., Wang, G. J., Tang, Y. W. & Song, X. Q. Photokinetic study on the photochromic reaction of aberchrome-540(tm) - a further comment about the use of aberchrome-540(tm) in chemical actinometry. *J. Photochem. Photobiol. A-Chem.* **88**, 31-34 (1995).

9 Heller, H. G. & Langan, J. R. Photochromic heterocyclic fulgides .3. The use of (e)-alpha-(2,5-dimethyl-3-furylethylidene) (isopropylidene)succinic anhydride as a simple convenient chemical actinometer. *J. Chem. Soc.-Perkin Trans. 2*, 341-343 (1981).
